# Gene Expression Changes in a Model Neuron Cell Line Exposed to Autoantibodies from Patients with Traumatic Brain Injury and/or Type 2 Diabetes

**DOI:** 10.1007/s12035-021-02428-4

**Published:** 2021-05-19

**Authors:** Mark B. Zimering, Vedad Delic, Bruce A. Citron

**Affiliations:** 1 Endocrine and Diabetes Section, Medical Service, VA New Jersey Healthcare System, 385 Tremont Ave, East Orange, NJ 07018 USA; 2 Robert Wood Johnson Medical School, New Brunswick, NJ USA; 3 Laboratory of Molecular Biology, VA New Jersey Health Care System, Research & Development (Mailstop 15), 385 Tremont Ave, East Orange, NJ 07018 USA; 4 Pharmacology, Physiology and Neuroscience, New Jersey Medical School, Rutgers University, Newark, NJ USA

**Keywords:** Neurodegeneration, Autoantibodies, Neurites, Cell death, RNAseq, Gene expression

## Abstract

Traumatic brain injury and adult type 2 diabetes mellitus are each associated with the late occurrence of accelerated cognitive decline and Parkinson’s disease through unknown mechanisms. Previously, we reported increased circulating agonist autoantibodies targeting the 5-hydroxytryptamine 2A receptor in plasma from subsets of Parkinson’s disease, dementia, and diabetic patients suffering with microvascular complications. Here, we use a model neuron, mouse neuroblastoma (N2A) cell line, to test messenger RNA expression changes following brief exposure to traumatic brain injury and/or type 2 diabetes mellitus plasma harboring agonist 5-hydroxytryptamine 2A receptor autoantibodies. We now report involvement of the mitochondrial dysfunction pathway and Parkinson’s disease pathways in autoantibody-induced gene expression changes occurring in neuroblastoma cells. Functional gene categories upregulated significantly included cell death, cytoskeleton-microtubule function, actin polymerization or depolymerization, regulation of cell oxidative stress, mitochondrial function, immune function, protein metabolism, and vesicle function. Gene categories significantly downregulated included microtubule function, cell adhesion, neurotransmitter release, dopamine metabolism synaptic plasticity, maintenance of neuronal differentiation, mitochondrial function, and cell signaling. Taken together, these results suggest that agonist 5-hydroxytryptamine receptor autoantibodies (which increase in Parkinson’s disease and other forms of neurodegeneration) mediate a coordinating program of gene expression changes in a model neuron which predispose to neuro-apoptosis and are linked to human neurodegenerative diseases pathways.

## Introduction

Traumatic brain injury (TBI) is a leading cause of long-term disability in US military combat veterans. Neuropsychiatric disorders and cognitive decline are “early” and “late” neurologic manifestations of exposure to “blast” and direct force brain injuries [1, 2]. While consequences of TBI have been linked to etiology of several neurodegenerative diseases, the most compelling evidence currently available is for a biological link between TBI and Parkinson’s disease (PD) [3]. PD is the most common of all the parkinsonian diseases which also include corticobasal degeneration syndrome, Dementia with Lewy bodies, multisystem atrophy, progressive supranuclear palsy, and vascular parkinsonism. These diseases are grouped together based on clinical presentation and pathological similarities which includes progressive neurodegeneration [4].

Vascular injury and inflammation may have an etiologic role in neuropsychiatric and neurodegenerative complications following TBI [5] in part via activation of humoral immune responses to antigens released following injury. One such putative antigen, the 5-hydroxytryptamine 2a receptor (5-HT2AR) is widely distributed in the central nervous system where it functions in the regulation of mood, thinking, perception, memory, sleep, and other important functions [6]. 5-HT2AR is a subtype of the 5-HT2R, a member of the G protein-coupled family of serotonin receptors [7]. Activation of 5-HT2AR is necessary for hallucinations associated which psychedelic drugs such as LSD which act as full or partial 5-HT2AR agonists [8]. In a recent report, long-acting agonist autoantibodies to the 5-HT2AR were increased in veterans suffering TBI in association with the late occurrence of neurodegenerative complications, e.g., dementia, Parkinson’s disease [9]. The long-acting agonist antibody to 5-HT2A receptor (in adult TBI, and type 2 diabetes mellitus without TBI) caused acute neurite retraction and accelerated cell loss in mouse neuroblastoma N2A cells via inositol triphosphate receptor-mediated cytosolic Ca^2+^ signaling [9, 10]. Normal Ca^2+^ is essential for helping orchestrate neuronal gene expression during development, as well as to maintain homeostasis which is altered following a TBI [11]. Neurons respond to insult and environmental stimuli by modulating Ca^2+^ release and reuptake from two major intracellular calcium stores endoplasmic reticulum and mitochondria [12–14]. Abnormal Ca^2+^ signaling may in turn lead up to gene expression changes that cause or contribute to buildup of toxic proteins (e.g., alpha synuclein, amyloid beta, Tau) and cause mitochondrial dysfunction resulting in neurodegeneration and cell death [3, 15].

Type 2 diabetes mellitus increases in aging veterans. We compared messenger RNA expression occurring downstream of 5-HT2A receptor activation in neuronal cells exposed to agonist 5-HT2AR autoantibodies isolated from plasma of traumatic brain injury patients with or without co-morbid type 2 diabetes and in type 2 diabetes without traumatic brain injury.

## Materials and Methods

### **Participants**

Participants were enrolled in local Veteran Affairs New Jersey Health Care System IRB-approved studies of the interaction between type 2 diabetes and traumatic brain injury in the late occurrence of neurodegeneration. All participants signed IRB-approved consent prior to blood drawing for autoantibodies isolation. Plasma was obtained in the morning.

All participants are males who suffered direct force head trauma. The time interval following TBI was variable ranging from 1 to 20 years or longer. Nearly all of the TBI patients (5 of 6) suffered a single, direct force mild TBI, i.e., concussion. None of the participants experienced blast injury. One of six TBI patients had experienced multiple mild TBIs which is expected to be associated with higher level and titer of harm-inducing autoantibodies [9]. The TBI patients were not selected on the basis of having had exposure to a particular type of TBI. The TBI patients were selected on the basis of harboring plasma 5-HT2AR binding autoantibodies that displayed 2.8-fold or higher binding *(vs.* background) in the ELISA. They were representative of men exposed to the most common type of TBI — a single mild direct force TBI [16] All diabetes patients had type 2 diabetes mellitus, and they were treated with a number of different oral anti-diabetic medications and/or insulin or incretin-based therapies.

### **Subgroups**

Each of four subgroups of participants was represented by three patients:

diabetes without harm-inducing plasma autoantibodies; diabetes harboring harm-inducing plasma autoantibodies without TBI, TBI alone with harm-inducing autoantibodies; and TBI + diabetes with harm-inducing autoantibodies.

### Protein-A Affinity Chromatography

Protein A affinity chromatography was performed as previously reported [17].

### Enzyme-Linked Immunoassay for 5-HT2A Receptor

The 5-HT2AR binding assay was carried out as previously reported [18] using (as capture antigen) an 18-meric linear synthetic peptide, Q….N, (LifeTein Inc., Hillsborough, NJ) having amino acid sequence identical to that of the second extracellular loop region of the human 5-HT2AR. A 1/25th dilution of the protein-A eluate fraction of plasmas was added to each well in duplicate. Autoantibody-antigen binding was detected with the use of peroxidase conjugated, goat-anti-human IgG (Sigma Chem Co, St Louis, MO) and appropriate substrate solution [18]. Color development was monitored at 490 nm using an IMark reader (Biorad).

### Cells

Mouse neuroblastoma N2A cells were obtained from American Type Culture Collection (Manassa, VA). Mouse neuroblastoma cells (N2A) were maintained in Dulbecco’s modified Eagle’s medium containing 10% fetal bovine serum. An identical number of cells was seeded into each of twelve T-75 cm^2^ flasks and incubated for 48 h until the cells had reached ~ 80% confluency. They were seeded at 2 × 10 ^6^ cells/ T-75 cm^2^ flask, and after 3 passages in culture (1:3 subcultivation ratio), each of twelve 80% confluent T-75 cm^2^ flasks contained ~ 5.6 × 10^6^ cells. Next a 1:25th dilution of each of the twelve protein A-eluate fractions of plasma was added to each flask of N2A cells (1 patient autoantibody/flask). The cells were incubated (in the presence of autoantibody) for 2 h at 37° C prior to RNA extraction.

### **Neurites**

The N2A neurite retraction assay was performed as previously reported [17]. Briefly, mouse neuroblastoma cells were seeded at low density in 35 mm dishes. After 24 h incubation to allow cell attachment and spontaneous neurite expression, a test protein-A eluate fraction of patient plasma was added (1:25 dilution) to dishes. Cells were observed over a 10-min time period for acute change in baseline neurite length. Percent basal neurite length represents the average of the (autoantibody stimulated/basal neurite length) in ten or more cells from two dishes, each cell expressing two or more neurites.

### **Cell Survival**

The measurement of accelerated mouse neuroblastoma cell loss was performed as previously reported [17]. Cell survival of N2A cells incubated for (12–16 h) with a 1:50th dilution of the protein-A eluate fraction of plasma was estimated using an MTT assay. Proportion of surviving cells represents the average of quadruplicate determinations compared to basal cell number in wells not containing test protein-A eluate fractions.

### **RNA Analysis**

RNA extraction was performed using the Rneasy Plus Mini Kit (QIAGEN cat. 74,134), according to the manufacturer’s specifications, on approximately 1 million N2A cells per flask.

For RNAseq, RNA samples were provided to the Rutgers Genomics Center, and they returned the sequences obtained. Briefly, the RNA integrity numbers were confirmed as greater than 7.0 with an Agilent TapeStation 2200, and poly(A) mRNAs were selected from the total RNA samples with oligo-d(T)25 magnetic beads (New England Biolabs). cDNA libraries were generated with the NEB next ultra RNAseq library kit and purified with AmpureXP beads and further characterized with an Agilent TapeStation and fluorescence (qubit) analysis. These libraries, specifically barcoded for each sample, were pooled in equimolar amounts and sequence for a 1 × 75 base read configuration with a NextSeq 500 instrument (Illumina, San Diego, CA). An average of 3.5 Gbases of sequence information was obtained per sample (corresponding to approximately 47 million sequences per sample).

### **Pathway Analysis**

CLC Genomics Workbench version 20.0, QIAGEN (Germantown, MD) was used to map the sequences obtained to the mouse genome and perform initial expression analyses. Ingenuity Pathway Analysis, QIAGEN (Germantown, MD) was used to identify pathways associated with genes dysregulated by patient autoantibodies.

### **Statistical Analysis**

Mean values are depicted ± standard deviation and were compared by ANOVA and Bonferroni post-hoc testing with P < 0.05 indicating significance.

## Results

### Autoantibodies in Subsets of TBI, T2DM and TBI + T2DM

Three subgroups of “test” patients were selected on the basis of having diagnoses of TBI with or without type 2 diabetes mellitus (T2DM) or T2DM without TBI (n = 3 in each subgroup) and who also harbored plasma autoantibodies which demonstrated high level of binding (3–3.5-fold background) to a linear synthetic peptide corresponding to the second extracellular loop of the human 5-HT2A receptor (Table 1). The control group consisted of age-matched patients with T2DM and without TBI (n = 3) whose plasma autoantibodies lacked significant binding to the 5-HT2A receptor linear synthetic peptide (Table 1). The incidence of late-onset sporadic form of PD increases in older adults with type 2 diabetes and following traumatic brain injury. Because of a previous association between PD and high level of autoantibody binding to the 5-HT2A receptor peptide [18], in the current study “test” patients were enriched by inclusion of 4/9 patients having PD vs. 0/3 “control” patients with PD (Table 1). Gene expression changes in response to 2-h incubation of mouse neuroblastoma (N2A) cells with a 1:25 dilution of patient autoantibodies was compared in “test” (n = 9) vs “control” (n = 3) patients who differed significantly in baseline autoantibody binding to the 5-HT2A receptor peptide.

**Table 1:** Baseline characteristics of the study participants

Group	N	Age (years)	5-HT2AR binding (AU)	No. with PD
Non-toxic DM	3	74.7 ± 9.0	0.06 ± 0.02	0
TBI	3	70.7 ± 6.1	0.14 ± 0.06	1
DM	3	62.0 ± 7.0	0.18 ± 0.03	2
TBI + DM	3	67.7 ± 8.1	0.15 ± 0.02	1

Results are mean ± SD; AU = arbitrary absorbance units (0.05 is the background level) in the ELISA employing a linear synthetic 18-meric peptide corresponding to the second extracellular loop of the 5-HT2AR receptor

### Effect of Diabetic and/or TBI Plasma Autoantibodies on Neuroblastoma Neurite Retraction or Cell Survival

**Fig. 1:**
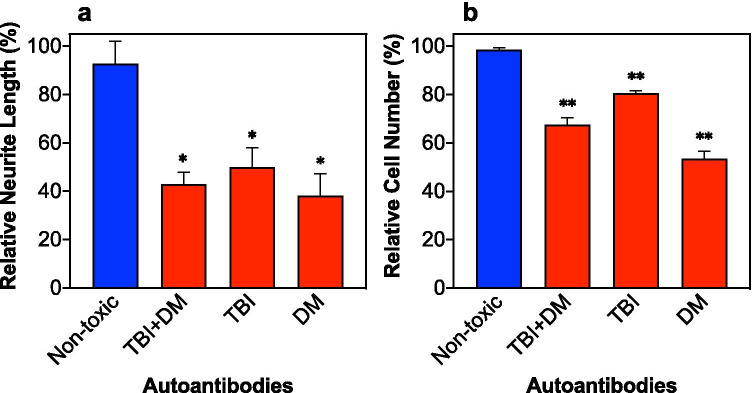
The effects of autoantibodies from TBI and type 2 diabetic patients on neuroblastoma cell health. **a** Acute neurite retraction of N2A model neurons was induced by autoantibodies from TBI and DM subsets. **b** Accelerated N2A cell loss from the autoantibodies. (N = 3 per group, *p < 0.01, **p < 0.001 vs. the nontoxic control antibodies)

As previously reported [18], autoantibodies in subsets of older adult type 2 DM, adult TBI, or T2DM + TBI which displayed increased binding to a linear synthetic peptide corresponding to the second extracellular loop domain of the human 5-HT2AR were neurotoxic. The length of the processes extended by the model neurons is an indicator of cellular health. As shown in Fig. 1a, exposure to the neurodegenerative autoantibodies caused significantly greater mean acute neurite length-shortening (i.e., retraction) in N2A mouse neuroblastoma cell vs nontoxic DM autoantibodies (P = 0.01) (Fig. 1a).

Cell loss was also monitored after incubation with the autoantibodies. The toxic autoantibody treatments promoted significantly greater mean accelerated loss in N2A neuroblastoma cells vs nontoxic DM autoantibodies (P = 0.001) (Fig. 1b).

### Gene Expression Changes in TBI + DM vs. Control Antibodies

**Fig. 2:**
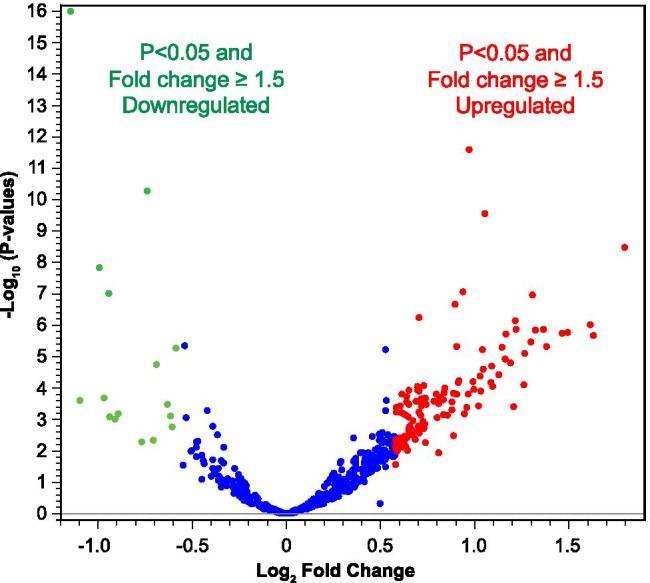
Statistical analysis of dysregulated genes. Gene expression changes in response to TBI + Db antibodies compared to control antibodies display mostly upregulated genes. A volcano plot illustrates the genes that displayed up (red) or down (green) regulation in excess of 1.5-fold and a statistically significant p value

A volcano plot (Fig. 2) illustrates the genes that displayed an up (red) or down (green) regulation in excess of 1.5-fold and a statistically significant p value in response to TBI + DM antibodies vs. control antibodies. A larger number of genes was significantly upregulated compared to genes significantly downregulated.

### Expression of Genes Affected by the Neurodegenerative Autoantibodies

**Fig. 3:**
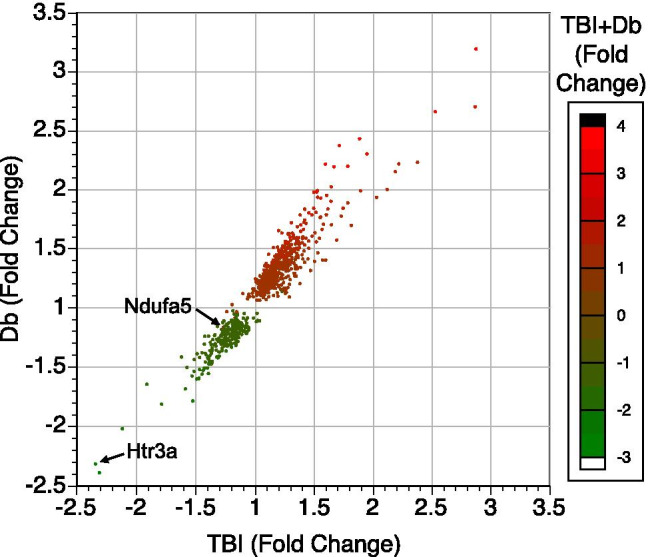
Expression of genes affected by the neurodegenerative autoantibodies. RNAseq mRNA values are shown for 806 genes and indicate gene expression fold changes of all of the test groups. Fold changes induced by the toxic TBI autoantibodies (x-axis), toxic Db autoantibodies (y-axis) and the TBI + Db autoantibodies (color scale). Each dot’s position and color represents the fold change seen relative to exposure to the control, non-toxic autoantibody samples. Some genes displayed discordance, e.g., Ndufa5 (NADH:ubiquinone oxidoreductase subunit A5) was downregulated 15–25% by TBI or Db samples, but was not downregulated by the TBI + Db samples and appears red. Htr3a was one of the most downregulated genes by autoantibodies from all 3 toxic conditions

Figure 3 depicts the fold changes induced by the autoantibodies from all three conditions with the x-axis representing fold changes from the TBI patient samples, the y-axis indicating the fold changes from the DM patient samples, and the color indicating the fold changes induced by the samples from the patients with TBI history and diabetes. This graph includes any gene where the expression levels from any condition met the expression cutoff of 50 rpkm and where the fold change for any sample group had a p value < 0.05. One can see that, with only some exceptions, any of the conditions that were experimentally found to negatively impact the health of the N2a model neurons produced relatively similar gene changes among the 25,000 mRNA sequences that were examined.

### Genes Up- or Downregulated by Autoantibodies in Plasma from TBI and/or DM Autoantibodies

Table 2 lists the genes significantly dysregulated by exposure to plasma autoantibodies from TBI and/or T2DM patients. Among the individual genes upregulated by autoantibodies in TBI and/or T2DM plasma are those encoding proteins having a role in ribosomal structure and function (RPl9-ps6, Rps14), programmed cell death (Pdcd10), cytotoxic T lymphocyte regulation (Ctla2a), tumor suppression(Rassf1), zinc finger encoded proteins (Znhit3), enzymatic suppression of apoptosis in resting lymphocytes (Dpp7), and B lymphocyte differentiation (Xlr3a) (Table 2). Functional categories comprising several or more individual genes that were upregulated significantly include: cell death, cholesterol biosynthesis, cytoskeleton-microtubule function and actin polymerization or depolymerization, regulation of cell oxidative stress, mitochondrial function, immune function, protein metabolism, and vesicle function (Table 2). Individual genes significantly downregulated by exposure to autoantibodies in TBI and/or T2DM include those having a role in microtubule function (MAP1B), cell adhesion (Nectin1, Ncam1, L1cam), neurotransmitter release or dopamine metabolism (synapsin II, complexin I, dopamine beta hydroxylase), flow-sensing in aortic endothelium (Gpr68), synaptic plasticity underlying learning and memory (Arc), maintenance of neuronal differentiation (nerve growth factor receptor), at least six genes involved in mitochondrial function (e.g., cytochrome b, and NADH dehydrogenase, subunit 4L (complex I)), and several genes (including G-protein coupled receptors) having a role in cell signaling: 5-hydroxytryptamine 3a receptor, G-protein coupled receptor kinase 3 (GRK3), and glypican 1- a cell surface heparan sulfate proteoglycan (Table 2).

**Table 2:** Genes dysregulated by exposure to autoantibodies affecting cellular health

Change direction	Fold changevs. control			
*Category*				
Gene symbol	Entrez gene name	TBI	DM	TBI/DM
Genes upregulated				
*Cell death*				
DDIT4	DNA damage inducible transcript 4	2.12	2.01	1.62
Pdcd10	Programmed cell death protein 10	1.85	2.3	3.23
Casp3	Caspase 3	1.27	1.34	1.3
Bnip3	Bcl2 interacting protein 3	1.56	1.98	2.69
Nae1	NEDD8 activating enzyme E1 subunit 1	NS	1.43	1.52
*Cholesterol biosynthesis*				
MSMO1	Methyl sterol monooxygenase 1	2.87	3.2	3.64
MVD	Mevalonate pyrophosphate decarboxylase	2.86	2.71	2.63
Cyp51	Cytochrome p450, family 51	1.89	1.99	2.3
Hmgcs1	3-hydroxy-3-methylglutaryl-CoA synthase	2.22	2.22	2.3
ACAT2	acetyl-CoA acetyltransferase 2	2.19	2.15	2.08
Insig1	insulin-induced gene 1	1.74	1.84	2.08
NSDHL	NAD(P) dependent steroid dehydrogenase-like	2.1	2.15	2.03
*Cytoskeleton*				
NME2	Nucleoside diphosphate kinase 2	1.78	2.2	3.07
TPPP3	Tubulin polymerization promoting protein family member 3	1.6	1.79	1.87
TUBA1A	Tubulin alpha 1 a	1.78	1.89	1.79
BRK1	BRICK1 subunit of SCAR/WAVE actin nucleating complex	NS	1.41	1.45
*Cell oxidation*				
GPX4	Glutathione peroxidase 4	1.55	1.77	1.87
GPX1	Glutathione peroxidase 1	1.52	1.56	1.57
PARK7	Parkinsonism associated deglycase	NS	NS	1.34
*Signaling/signal transduction*				
KLF6	Kruppel like factor 6 transcription factor	2.37	2.12	2.22
PPP1R35	Protein phosphatase 1 regulatory subunit 35	1.69	1.99	2.09
BEX4	Brain expressed X-linked 4	1.65	1.66	2.06
TRIB3	Tribbles pseudokinase 3	2.17	1.91	1.95
GDF15	Growth diff factor 15	1.93	1.72	1.72
CD63	Cd63 antigen	1.51	1.64	1.66
RABAC1	Rab acceptor 1	1.45	1.57	1.63
RACK1	Receptor for activated C kinase 1	1.37	1.56	1.65
*Mitochondrial function*				
Ndufb8	NADH dehydrogenase 1 beta subunit 8	1.53	1.94	2.25
Ndufaf6	NDH: ubiquinone oxidoreductase complex	1.51	1.98	2.2
Fmc1	Formation of mitochondrial complex V assembly factor 1 homolog	1.51	1.81	2.01
Atp5e	ATP synthase, H + transporting mitochondrial F1 complex, epsilon subunit	1.41	1.72	2.32
Apt5mg	ATP synthase membrane subunit g	1.37	1.54	2.12
*Immune function*				
BRI3	Brain protein I3	1.56	1.79	1.94
CTL2a	Cytotoxic T lymphocyte associated protein 2 alpha	2	2.25	2.91
CREG1	Cellular repressor of E1A stimulated genes 1	1.68	1.76	1.84
*Protein metabolism*				
TOMM5	Translocase of outer mitochondrial membrane	2.03	1.64	2.46
PSMA6	Proteasome 20S subunit alpha 6	1.39	1.7	2.13
*Vesicle function*				
NAP1L5	NSF attachment protein alpha	1.63	1.62	1.51
Genes downregulated				
*Signaling/signal transduction*				
HTR3A	5-hydroxytryptamine 3A receptor	− 2.34	− 2.31	− 2.73
RET	Ret proto-oncogene	− 1.79	− 1.81	− 1.92
GPC1	Glypican	− 1.52	− 1.53	− 1.69
SRF	Serum response factor	− 1.69	− 1.59	− 1.63
GPR69	G protein coupled receptor 68	− 1.52	− 1.63	− 1.76
GRK3	G protein coupled receptor kinase 3	− 1.59	− 1.66	− 1.95
*Cytoskeleton*				
TLN2	Talin 2	− 1.64	− 1.7	− 1.74
MAP1B	Microtubule associated protein 1B	− 1.4	− 1.52	− 1.51
ACTG1	Actin gamma 1	− 1.47	− 1.38	− 1.42
*Extracellular matrix*				
NCAM1	Neural cell adhesion molecule 1	− 1.71	− 1.7	− 1.71
L1CAM	L1 cell adhesion molecule	− 1.58	− 1.66	− 1.71
Nectin 1	Nectin cell adhesion molecule 1	− 1.76	− 1.79	− 1.77
*Neurotransmitter release/synthesis*				
SYN2	Synapsin II	− 1.56	− 1.71	− 2.23
DBH	Dopamine beta hydroxylase	− 2.31	− 2.39	− 2.69
CPLX1	Complexin I	− 1.43	− 1.51	− 1.67
*Neuronal differentiation*				
PLXNA4	PlexinA4	− 1.65	− 2.07	− 2.7
NGFR	Nerve growth factor receptor	− 1.39	− 1.47	− 1.61
*Mitochondrial function*				
MT-ND4L	NADH dehydrogenase, subunit 4L (complex I)	− 1.53	− 1.79	− 2.13
MT-ND4L	NADH dehydrogenase, subunit 4 (complex I)	− 1.47	− 1.59	− 1.91
MT-ND5	NADH dehydrogenase, subunit 5 (complex I)	− 1.44	− 1.53	− 1.87
MT-CYB	Cytochrome B	− 1.45	− 1.56	− 1.85
MT-ND6	NADH dehydrogenase, subunit 6 (complex I)	− 1.4	− 1.42	− 1.63
MT-ND1	NADH dehydrogenase, subunit 1 (complex I)	NS	NS	− 1.46

Upregulated and downregulated genes after exposure to autoantibodies from patients with traumatic brain injury and/or type 2 diabetes. *NS* not significant, i.e., p ≥ 0.05 vs. control

### Effect of TBI and/or DM Autoantibodies on Gene Expression in Serotonergic Receptors

The mammalian 5-HT2A receptor is highly expressed in brain regions important in the regulation of mood, thinking, perception, sleep, and feeding behaviors [6]. In many of the same brain regions, other members of the 5-HT receptor family (total 14 isoforms) are also expressed and may have coordinate or antagonistic functions as those of the 5-HT2A receptor. Of interest, only the Htr2a and Htr3a genes were significantly expressed (i.e., RPKM > 50) in N2A mouse neuroblastoma cells, and expression of Htr2a was not altered following exposure to TBI and/or DM autoantibodies. The Htr3a gene displayed significant downregulation across all three test subgroups vs control, and a significant p value (Table 2). Htr3A is the only member of the 5-HT receptor family which encodes an ion channel [19]. 5htr3a co-localizes with AchR on presynaptic terminals in the striatum [20]. The significant downregulation of 5htr3a by the test group patient autoantibodies vs control is interesting since 5htr3a normally functions to increase dopamine neurotransmitter release in the striatum [20]. Thus, decreased expression of the 5htr3a receptor (in response to TBI and T2DM autoantibodies) may interfere with dopamine release in the striatum- a hallmark of Parkinson’s disease.

A striking feature of the autoantibodies effect on serotonin 2A receptor-mediated neurite retraction in neuroblastoma cells was the previously reported finding [17] that the phenotypic response did not undergo “rapid desensitization” which is typical of G-protein coupled receptor signaling. Of interest, expression of G-protein receptor kinase 3 (Grk3) which normally functions in mediating desensitization of G-protein coupled signaling [21] was significantly downregulated (in N2A cells) by the TBI and/or T2DM autoantibodies.

### Gene Ontology Analysis

Gene ontology revealed several processes underlying the gene expression changes in response to autoantibodies in TBI and/or T2DM. First, the “neuron apoptosis process” included four genes which were significantly upregulated in N2a cells treated with test patient autoantibodies including Casp3, Bnip3, Nae1, and Gpx1. In a prior report, diabetic plasma autoantibodies in patients having microvascular complications caused endothelial cell apoptosis via activation of caspase 3 [22], an important mediator of apoptosis. Bnip3 is a member of the apoptotic Bcl-2 protein family which plays a role in permeabilization of the outer mitochondrial membrane. NEDD8 activating enzyme E1 subunit 1 (product of the Nae1 gene) is involved in neddylation — a process in which the ubiquitin-like protein NEDD8 is covalently bound to proteins. Neddylation has been implicated in Alzheimer’s dementia by promoting neuron apoptosis via promotion of cell cycle entry [23]. Taken together, accelerated neuroblastoma cell loss following exposure to autoantibodies in diabetes, TBI and TBI plus DM from patients having co-morbid Parkinson’s disease, or dementia [10] may be mediated by neuroapoptosis involving (in part) increased Casp3, Bnip3 and Nae1 gene expression. Glutathione peroxidase 1 (Gpx1) protects the cell from oxidative damage. Significant upregulation of gene expression in Gpx1 and in proteasome subunits alpha 1 (Psma1) and beta type 4 (PSMB4) suggests likely involvement of the ‘negative regulation of inflammatory response to antigenic stimulus’ process. The “response to nerve growth factor” process is suggested by significantly decreased expression of complexin-2 (Cplx2) — a cytosolic protein involved in synaptic vesicle exocytosis. Decreased expression of Cplx2 is associated with schizophrenia and Huntington’s disease [24] and with decreased neurotransmitter release [25]. Finally, significant upregulation of the gene Zfand2b which encodes a zinc finger type protein suggests additional involvement of the “regulation of insulin like growth factor receptor signaling pathway.” Zfand2b protein product interacts with the proteasome in mediating proteostasis via binding to and helping to eliminate misfolded proteins from the cell.

### Signaling Pathways Affected by Degenerative Autoantibodies

Compared to control exposures, the harm-inducing autoantibodies caused changes in several pathways. Focusing on the changes induced by TBI + Db subgroup autoantibodies, pathways that seemed biologically relevant included mitochondrial dysfunction pathway and Parkinson’s disease pathway. Upstream regulators that hit statistically and seemed biologically interesting included NFE2L2 (Nrf2) upstream network. Of note, the Parkinson’s disease pathway includes dysregulation of caspase 3 and Park7 — two genes which were significantly upregulated following exposure to TBI + DM autoantibodies (Table 2) plus some genes that did not meet all criteria but were identified as statistical hits in the pathway analysis. These included upregulation of UCHL1 (involved in Lewy body formation) and Cyc5 (can activate caspase cascades). In this pathway, the only discrepancy was p38 MAPK that can upregulate Cyc5 and was expected to be upregulated; however it was found downregulated. Genetic factors which block dopamine release lead to dopamine accumulation and the production of reactive oxygen species, a component of the Parkinson’s disease pathway. Three genes significantly downregulated by harm-inducing autoantibodies (synapsin II, complexin I, and htr3a) mediate neurotransmitter release, and a fourth downregulated gene, dopamine beta hydroxylase, metabolizes dopamine to norepinephrine. Taken together, their combined significant downregulation may contribute to dopamine accumulation. Dopamine is normally metabolized to H202 which inhibits mitochondrial complex I function — a key target in the mitochondrial dysfunction pathway. Evidence in favor of involvement of the mitochondrial dysfunction pathway is significant downregulation of five genes belonging to mitochondrial complex I by the harm-inducing autoantibodies (Table 2). Finally, a number of different genes significantly dysregulated by the harm-inducing autoantibodies provide linkage(s) to the Nrf2 upstream network including Syn2, Rack1, BNIP3, CREG1, Gpx1, L1CAM, ACTG1, PSMA6, and other genes encoding subunits of the proteasome (Table 2).

### Overlapping Effects of Autoantibodies from Different Neurodegenerative Conditions

**Fig. 4:**
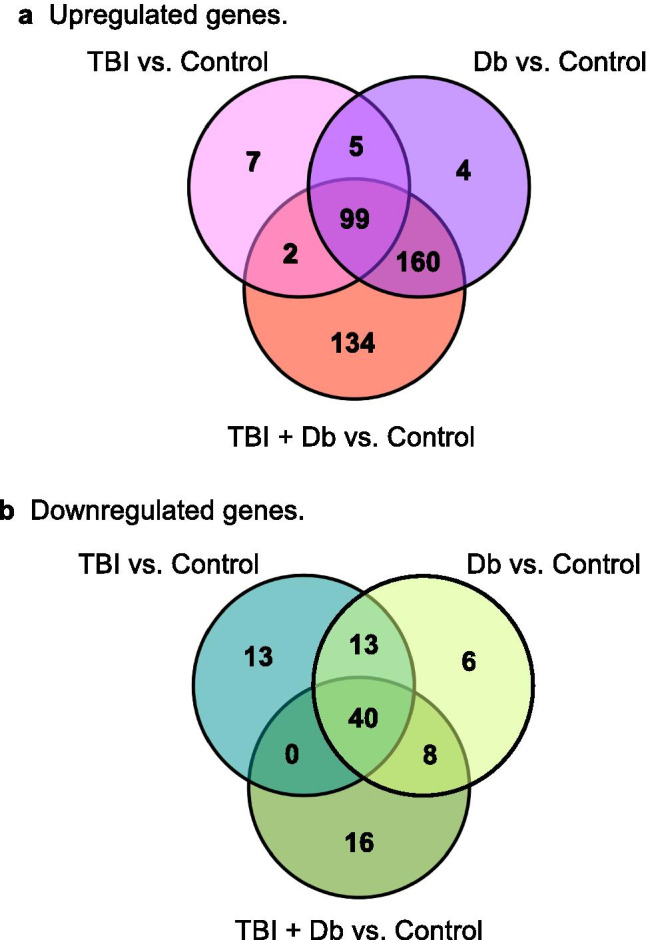
Overlapping effects of autoantibodies from different neurodegenerative conditions. The Venn diagram indicates genes significantly up- (**a**) or downregulated (**b**) by TBI and T2DM 5-HT2AR binding plasma autoantibodies

The Venn diagram indicates genes significantly up- or downregulated by TBI and T2DM 5-HT2AR binding plasma autoantibodies (Fig. 4). There was significant overlap in gene dysregulation among the three subgroups vs control. For example, a total of 99 genes were significantly upregulated in all three “test” subgroups compared to control (Fig. 4a). An additional 160 genes were significantly upregulated in both TBI + Db and Db subgroups, but not in TBI alone. In contrast, only 2 genes were upregulated in both TBI + Db and TBI, but not in Db (Fig. 4a). TBI + Db had the highest number of significantly upregulated genes (395) followed by Db alone (268) and TBI alone (113). Total 64 genes were significantly downregulated in TBI + Db antibodies compared to 66 genes in TBI and 67 genes in Db (Fig. 4b). Forty genes were significantly downregulated in all three subgroups vs control (Fig. 4b).

### Mitochondrial and Cell Oxidation Gene Expression Changes

Genes associated with cell oxidation were found to increase in excess of 1.5 fold in TBI, DM and TBI/DM, with TBI/DM generally having slightly higher expression fold change among cell oxidation genes for glutathione peroxidase 4 and 1. Parkinsonism associated deglycase gene was the only cell oxidation gene expression significantly elevated (1.34-fold) in TBI/DM group compared to control.

In general, genes corresponding to the mitochondrial electron transport chain complex I subunits were found to decrease, while subunits corresponding to mitochondrial complex V (ATP synthase) were found to be upregulated. Both proportion of up- and downregulation followed an increasing trend with TBI/DM having greatest increase followed by DM and TBI groups compared to controls. Translocase of outer mitochondrial membrane (TOMM5) was also found to increase 2.46-fold in TBI/DM, 2.03-fold in TIB, and 1.64-fold in DM groups compared to controls.

## Discussion

These are the first data suggesting that long-lasting agonist 5-HT2A receptor-targeting autoantibodies in plasma from patients having TBI and/or T2DM and suffering with microvascular and neurodegenerative (e.g., PD) complications cause significant modulation of expression in genes underlying neuroapoptosis, the maintenance of differentiated neuronal morphology, neurotransmitter release, synaptic plasticity, and amyloid beta precursor protein interactions. The present findings are consistent with our prior reports that serotonin-2A receptor binding autoantibodies prevalent in 85% (17/20) of older adults with sporadic Parkinson’s disease [10] is not merely a “bystander phenomenon.” The autoantibodies orchestrate a coordinated program of gene expression changes in neuronal cells resulting in decreased neuronal differentiation and decreased neuron survival consistent with the observed acute effects of the autoantibodies in neuroblastoma cells in vitro [10, 18]. These data are the first to suggest that 5-HT2A receptor activation in neuroblastoma cells causes significant downregulation in the 5ht3A gene — which encodes a ligand-gated ionic channel important in mediating fast depolarizing responses underlying dopamine release in the striatum [20]. The 5ht3a gene is also expressed in the gut and has a role in intestinal motility [26]. Dysregulating gastrointestinal motility is a common complication in patients suffering with Parkinson’s disease.

G-protein coupled receptor kinase 3 (Grk3) was originally reported as the beta- adrenergic receptor kinase-2 (BARK) which phosphorylates the ligand-occupied beta adrenergic receptor leading to beta-arrestin-mediated receptor desensitization [21]. In recent studies, the GRK3 gene isoform was reported to be preferentially highly expressed in olfactory cilia where it functions in desensitization of odorant-induced G-protein coupled receptor signaling [27]. GRK3 knockout mice had altered fast second messenger formation and impaired desensitization following odorant-induced G-protein coupled receptor activation [28]. More study is needed to determine whether downregulation of GRK3 gene by autoantibodies from PD patients may play a role in dysregulated olfaction common in human neurodegenerative disorders [29].

Complex I is the largest of the electron transport chain complexes and is the site of most ROS production. Dysfunction in complex I is closely associated with PD [30]. In an effort to decrease ROS, cells might lower expression of complex I subunits. To increase the flux of electrons down the electron transport chain and thereby limiting the chance for free radical formation, cells may increase expression of ATP synthase subunits. Increase of ATP synthase activity would prevent formation of free radicals by limiting electrons from straying resulting in oxygen molecules with unpaired electron valence shells. Most reactive oxygen species production occurs in the mitochondria and therefore, mitochondrial ROS production would be the main driver of increased cell oxidation responsive genes which include glutathione peroxidase 1 and 4. Parkinsonism associated deglycase (more commonly known as DJ-1 or PARK7) gene expression was only significantly increased in TBI/DM group suggesting small synergistic effect. DJ-1 normal function is to provide neuroprotection, and loss of function mutations in DJ-1 are a known cause of familial PD [31, 32].

The level of 5-HT2A receptor targeting autoantibodies in plasma from either adult obese type 2 diabetes mellitus or TBI alone was previously reported to be significantly associated with systemic inflammation (i.e., WBC) [9]. The present finding that the number of upregulated genes in N2A cells exposed to neurodegenerative autoantibodies was highest in (TBI + Db) compared to (Db without TBI) or (TBI alone) may be consistent with chronic inflammation arising from more than one source in (TBI + Db). In support of this possibility, the mean baseline WBC was nearly significantly higher in (TBI + Db) vs. (Db alone): 12.8 ± 1.8 vs 9.2 ± 0.5 K/cm^3^; p = 0.05. Baseline WBC was more than two-fold higher in (TBI + Db) compared to (Db without harm-inducing autoantibodies): 12.8 ± 1.8 vs. 6.4 ± 1.3 K/cm^3^; p = 0.015). The clinical relevance of this observation is that lifetime TBI-sufferers are likely to benefit from lifestyle modifications (diet, exercise) that promote maintenance of ideal body weight and lower the risk of future development of obese type 2 diabetes mellitus. In a prior retrospective study, higher body mass index was a significant predictor of the post-TBI occurrence of a composite neurodegenerative disease outcome in eighty middle-aged and older adults [33]. Treatment of type 2 diabetes mellitus which results in long-term improved glycemic control could potentially affect autoantibody levels since it is expected to reduce the risk of microvascular complications. Microvascular diabetic complications were significantly associated with an increased prevalence of harm-inducing 5-HT2AR targeting autoantibodies in an adult type 2 diabetes mellitus population [18].

There are several limitations to the findings in the present study. First, the use of mouse neuroblastoma N2A cells may affect the generalizability of the findings to adult neurons. Future work should consider comparing these responses to those in primary neurons. Second, all study patients were men who suffered from mild TBI. It is not known whether more severe grade of TBI injury or inclusion of women having mild TBI would result in a different pattern or large number of neurodegenerative gene expression changes.

In summary, RNA seq of neuroblastoma cells exposed to test TBI and T2DM autoantibodies having increased binding to a 5-HT2A receptor peptide involved in receptor activation confirmed involvement of neuron apoptosis in mediating accelerating N2A cell loss downstream of long-lasting receptor activation. It also suggests possible involvement of a number of different genes involved in neurotransmitter release and the regulation of G-protein coupled receptor signaling as candidate mediators of important neurobiological and cardiovascular responses previously associated with increased level of the autoantibodies.

## Data Availability

All data generated or analyzed during this study are available from the corresponding author on reasonable request. The RNAseq data will be available online in the NCBI GEO.

## Declarations

### Disclaimer

The contents do not represent the views of the Department of Veterans Affairs or the US Government, and the opinions, interpretations, conclusions, and recommendations are those of the authors and are not necessarily endorsed by the Department of Defense.
